# Attitudes and Opinions of Parents towards Water-Only Drink Policy at Junior Triathlon Events

**DOI:** 10.3390/ijerph19148529

**Published:** 2022-07-12

**Authors:** Brooke L. Devlin, Kiera J. Staley, Gina L. Trakman, Adrienne K. Forsyth, Matthew G. Nicholson, Grant Cosgriff, Melanie Chisholm, Regina Belski

**Affiliations:** 1 Department of Sport, Exercise and Nutrition Sciences, La Trobe University, Melbourne, VIC 3086, Australia; g.trakman@latrobe.edu.au (G.L.T.); regina.belski@latrobe.edu.au or r.belski@latrobe.edu.au (R.B.); 2 Centre for Sport and Social Impact, La Trobe University, Melbourne, VIC 3086, Australiam.nicholson@latrobe.edu.au or matthew.nicholson@monash.edu (M.G.N.); 3 School of Human Movement and Nutrition Sciences, University of Queensland, Brisbane, QLD 4072, Australia; 4 School of Behavioural and Health Sciences, Australian Catholic University, Melbourne, VIC 3065, Australia; 5 Monash University Malaysia, Kuala Lumpur 47500, Malaysia; 6 Triathlon Victoria, Melbourne, VIC 3206, Australia; grant.cosgriff@ausport.gov.au; 7 Victorian Health Promotion Foundation (VicHealth), West Melbourne, VIC 3003, Australia; mchisholm777@gmail.com

**Keywords:** sugar-sweetened beverages, hydration, adolescents, junior athletes

## Abstract

Rates of childhood obesity within Australia continue to rise, with consumption of sugar-sweetened beverages one contributing factor. Community sport provides an opportunity to implement policies promoting water as the beverage of choice. However, the attitudes of parents toward a water-only policy are not known. This cross-sectional study aimed to investigate parents’ opinions towards beverage consumption and a water-only policy. Data were collected from participants (n = 159) using an investigator-designed questionnaire, administered using iPads, at a junior Triathlon Victoria event. Water was the most popular beverage provided before (75%), during (85%) and after (61%) sport. Parents were more likely to provide sports drinks to children older than 14 years (27%). Three-quarters (77%) of parents reported having received no information regarding hydration requirements. Parents rated the importance of hydration prior to, during and after a triathlon as high (9.08 ± 1.2, 8.76 ± 1.3 and 9.30 ± 0.4 out of 10, respectively). Parents were supportive of a water-only policy at all junior triathlon events and all junior sporting events (7.94 ± 1.3 and 7.86 ± 1.9, respectively). There was less support for a water-only policy for adult triathlons (6.40 ± 3.1). A water-only drink policy at junior sport is viewed positively by parents. This warrants further research and policy development to facilitate behaviour change.

## 1. Introduction

Overweight and obesity is an increasing concern for children and adolescents within Australia, with one in four children and adolescents aged 2 to 17 years classed as overweight or obese in 2018 [1,2]. Furthermore, children and adolescents are significantly more likely to be overweight or obese at ages 10 to 17 than those of the same age two decades earlier [1]. Consumption of sugar-sweetened beverages, such as sports drinks and juices, contribute to increasing rates of overweight and obesity and associated health concerns [3,4]. The Victorian Health Promotion Foundation (VicHealth), in the state of Victoria, Australia, aspires to positively influence the behaviours of Victorians related to choosing water as a beverage of choice rather than sugar-sweetened beverages. Additionally, they aim to create healthier sporting environments and settings by reducing the availability and access to sugar-sweetened beverages in sporting environments and at events for children.

In 2019, up to 60% of Australian children regularly participated in organised physical activity outside of school hours, and approximately 72% of these children did sport-related activities [5]. In recent years, interest and participation in triathlons consisting of three different events: swimming, cycling and running, has grown. Specifically, Triathlon Australia offers a TRYstars program to introduce school age children to triathlons for the first time and promote a positive experience that leads to long-term engagement with the sport. Rates of participation in junior triathlon events in Australia are high and growing. In 2018, approximately 35,000 children participated, which was an increase of approximately 10,000 from the year prior [6]. This level of engagement and participation is exciting from a public health perspective as physical activity participation helps combat issues of overweight/obesity in children and adolescents [7,8]. Despite this increasing involvement in physical activity and sports outside of school hours, rates of overweight/obesity continue to rise [1,2]. Therefore, consideration of other health-related behaviours that can be promoted via community sport, such as reducing the consumption of sugar-sweetened beverages, is warranted. Policies surrounding foods and beverages that are both available and consumed at sporting events/centres are strategies that could be considered.

Australian sporting organisations at both the national level (i.e., national sports organisations) and the state level (i.e., states’ sports associations such as Triathlon Victoria) are only just beginning to consider the need for policies regarding the provision of beverages and healthy food options available at sporting events. A study of 108 sports clubs in New South Wales, Australia, revealed that only 3 sporting organisations had a written policy regarding healthy eating and fluids, with only a small portion of clubs encouraging water-only consumption (i.e., water as the beverage of choice) [9]. Water-only strategies and policies have recently gained significant attention, particularly in the school sector, with many schools in New Zealand recently adopting a water-only school policy [10] and some regions in Australia heavily promoting water-only schools and workplaces [11]. Adoption of water-only sporting environments and events has not yet received wide attention or implementation. However, VicHealth is one organisation aiming to promote water as the beverage of choice through initiatives such as the Water in Sport initiative [12]. The Water in Sport initiative 2018–2020 was an investment under the VicHealth Healthy Eating Strategy 2017–2019, which sought to improve the availability and promotion of water instead of sugary drinks in sports and recreation facilities. This initiative built on the insights and outcomes from previous VicHealth investments that aimed to influence water as the beverage of choice in sporting environments.

At the time of this study, Triathlon Victoria did not have a policy regarding encouraging water consumption or making water the beverage of choice. Of note, triathlons typically take place in the summer/warmer months. Fluid consumption and hydration are of critical importance during this time of year and children are likely to be thirsty and seek beverages to hydrate. Within a triathlon event, there are numerous opportunities to provide children with beverages: before, during and after an event, to encourage hydration and promote water as the beverage of choice. Typically, junior triathlon events are completed in a short timeframe (i.e., under 60 min) and water is an appropriate choice to maintain hydration in junior athletes, without the need of other beverages such as sports drinks [13,14].

Implementing public health promotion policies in sporting environments is challenging. This is mainly due to funding and a lack of support, as there is limited research exploring the long-term impact of such health promotion policies on the health behaviours of the Australian population. However, the sporting sector, in this case the triathlon event environment, provides an opportunity to promote a healthy lifestyle behaviour. This warrants exploration into the accessibility and availability of beverages, and the resulting impact on consumption behaviours and health. Parents’ opinions and support of policies within sporting environments is important to ensure they uphold and agree with the policy principles and application to drive successful implementation. The current practices, as well as opinions and attitudes of parents towards beverage consumption and a water-only policy within Triathlon Victoria event settings, is not known. Parents’ opinions and attitudes are likely to be important because parental receptivity towards policies in junior sport is likely to influence the policy’s efficacy. Therefore, the primary aims of this study were to investigate parents’ attitudes and opinions towards a water-only policy and explore beverage consumption during a junior triathlon event.

## 2. Materials and Methods

### 2.1. Study Design

This cross-sectional study was part of a larger Victorian Health Promotion Foundation (VicHealth) project to promote and encourage water as the beverage of choice for Victorians. Triathlon Victoria, in partnership with VicHealth, aimed to explore parent opinions of beverage consumption for children participating in a Triathlon Victoria event by administering a questionnaire at one Triathlon Victoria Junior Event (March 2017). This study was approved by the Human Research Ethics Committee and all participants gave implied consent by completing the questionnaire.

Data were collected at one Triathlon Victoria event held in March 2017 in Melbourne, Victoria. The junior triathlon event of interest contained three different events (Kids, Mini and Sprint) and five different age group categories as defined by Triathlon Victoria (Table 1). The questionnaire was administered to parents/guardians of children participating at the event using iPads. Volunteers appointed by Triathlon Victoria distributed the iPads at the event for completion of the questionnaire.

### 2.2. Participants

Parents/guardians of children competing in the Triathlon Victoria event were invited to complete the questionnaire. The event involved both male and female children and age categories from 7 through to 19 years of age (Table 1). Approximately 350 children took part in the triathlon event and a total of 159 parents completed the questionnaire over 1 day.

### 2.3. Study Tool: Questionnaire

An investigator-designed questionnaire (Table 2) was used to survey parents/guardians to gain insight into the knowledge of hydration and beverage choices for their children in association with triathlons, and to explore their opinions towards a water-only policy for Triathlon Victoria. Details were also obtained about the number of children the parents had participating and the specific event they were taking part in on the day of data collection.

### 2.4. Statistics

Statistical analysis was conducted using IBM SPSS Statistics for Windows, Version 22.0 (IBM Corp., Armonk, NY, USA, 2013), with significance set at *p* ≤ 0.05. Statistics are presented as proportions (%), means and standard deviations unless otherwise specified. All questionnaire items were analysed, open-ended and qualitative responses were analysed through grouping by theme/category and the number of comparable responses within a theme/category were summed and reported. Where responses were compared based on age, participants were categorised as <14 years vs. ≥14 years. This was determined as the age cut-off where parents have less influence on their children’s drink choices.

## 3. Results

### 3.1. Participant Characteristics

There were 159 parents who completed the questionnaire. A total of 129 parents (81%) had 1 child participating, 25 (16%) had 2 children participating and 5 parents (3%) had 3 children participating. Children participating ranged in age from 7 through to 19 years of age. Of the children participating, 20% were in the 7–9-year-old ‘kids triathlon’ category, 28% were in the 10–14-year-old ‘kids triathlon’ category, 44% in 12–19-year-old ‘mini triathlon’ category, 3% in the 14–15-year-old ‘sprint triathlon’ category and 5% in the 16–19-year-old ‘sprint triathlon’ category (as per Table 1). Whilst over the age of 18 is not considered ‘junior’, this age group aligns internationally with ‘junior’ for triathlon.

### 3.2. Current Practices

**Figure 1:**
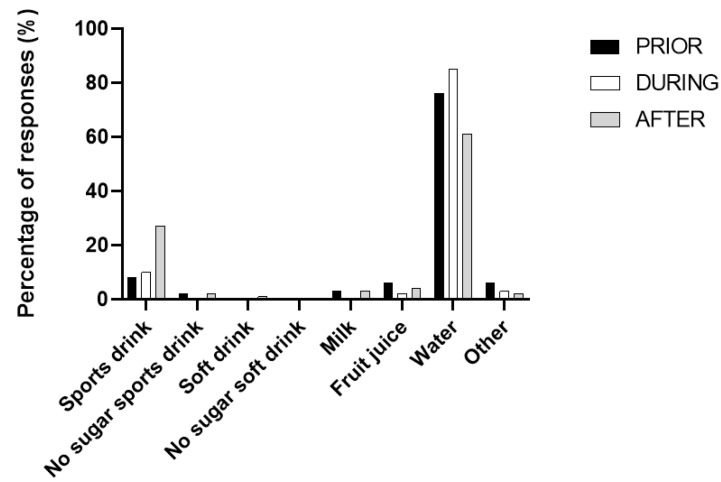
Parental reported practices of drink provision to children prior to, during and after a triathlon event.

Water was the most popular beverage reported to be provided to junior athletes, before (75%), during (85%) and after (61%) sport (Figure 1). Parents reported more sports drinks being provided to older participants (>14 years) than younger participants, especially after sport, with 27% of parents overall reporting providing a sports drink to their child after sport. Separated by group, this was 60% of parents with children in the 14 to 15-year-old group and 55% of parents with children in the 16–19-year-old group, compared to 18% in the 7 to 9-year-old group.

### 3.3. Current Knowledge and Previous Education

Almost all (99%) of the parents agreed with the statement “After an event, children should be encouraged to have a drink of water”. The majority (77%) of respondents reported having received no information regarding the hydration requirements of children at sporting events. Of those who had received information regarding the hydration requirements of children at sporting events (23%), only 14% reported making a change or contemplating making a change based on the information, and 50% reported that it “confirmed what I was doing was right”.

### 3.4. Importance of Hydration

**Figure 2:**
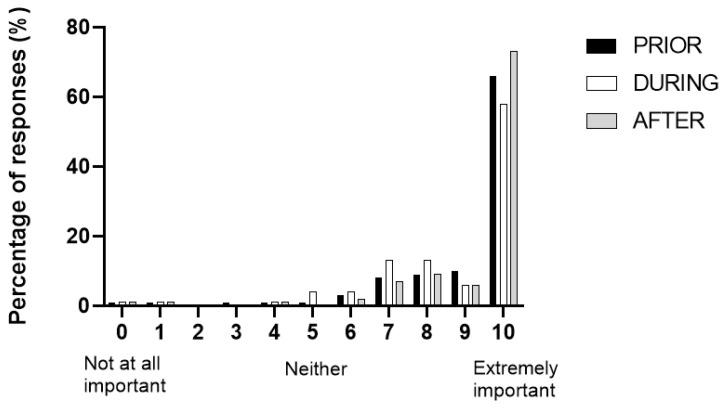
Ratings of importance of hydration prior to, during and after a triathlon event by parents of children participating in a Triathlon Victoria event in Melbourne.

Parents rated the importance of hydration prior to, during and after triathlon events as high, with mean responses of 9.08 ± 1.2, 8.76 ± 1.3 and 9.30 ± 0.4, on the 0–10 Likert scale (0 = not at all important, 10 = extremely important), respectively. The overall percentages of responses to each question (prior to, during and after) are presented in Figure 2.

### 3.5. Opinions on ‘Water-Only’ Policy

**Figure 3:**
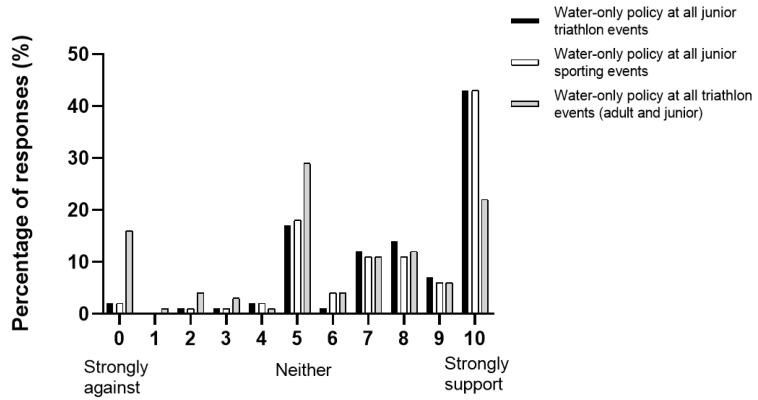
Opinion of parents with children participating in a triathlon event towards water-only policy for triathlon and junior sporting events.

Parents were supportive of a water-only policy at junior triathlon events (Figure 3). The mean response to support a water-only policy at all junior triathlon events and all junior sporting events on the 0–10 Likert scale was 7.94 ± 1.3 and 7.86 ± 1.9, respectively. However, the mean response for support for a water-only policy at both adult and junior triathlon events was lower, at 6.40 ± 3.1.

The parents with children in the 14–15- and 16–19-year-old groups were the least supportive of a water-only policy. The participants’ opinions were split regarding the suitability of a water-only policy for all (adult and junior) triathlon events (55% in support, 29% unsure and 16% not supportive).

## 4. Discussion

To our knowledge, this is the first study to explore attitudes and opinions of parents towards beverage consumption during a junior triathlon event and to obtain parent opinions towards a water-only policy for junior sporting events. The key findings were: (1) water is the main beverage reported to be provided and encouraged by parents before, during and after triathlon events, (2) older children are more likely to be provided sports drinks by their parents, (3) minimal prior education focusing on hydration and beverages for children in and around sporting events has previously been provided to parents and (4) parents of children participating in junior triathlons strongly supported a policy-based initiative focused on water-only junior triathlon and other junior sporting events; however, there was less support for a water-only policy at all triathlon (junior and senior) events.

VicHealth promote and encourage water as the beverage of choice for both children and adults within Victoria, Australia, to reduce the consumption of sugar-sweetened beverages and the negative health effects associated with these products. It was found in this study that water was the most popular beverage of choice reported by parents to be provided to children before, during and after sport. Access to water before, during and after sport is a better and more affordable option and children do not need sports drinks for hydration or energy if adequate food intake is provided before and after the event [13,14].

Our results demonstrate that parents are more likely to provide sports drinks after triathlon events for older children, with more parents providing sports drinks in the 14–15- and 16–19-year-old age groups compared to 7–9-year-old age groups. This indicates that parents may consider sports drinks more acceptable or necessary for older children. As children enter their teenage years, they have increased independence and their total daily energy requirements increase. Despite this, water-only is still sufficient as a rehydration beverage in this age group. Parents may not be aware of this, and children may be influenced by the strategic marketing of sports drink products and increased peer pressure of teenage years, thereby seeing sports drinks as a superior way to refuel and replenish. There may also be a misconception that teenagers require sports drinks to replenish after an event, and if this is the case, it provides another reason to educate both parents and teenagers alike regarding hydration needs.

A large proportion of parents reported having never received any education about hydration practices for sporting events. Despite many parents providing water, education focusing on children’s hydration requirements and needs as they progress into the teenage years could further reduce the provision of sports drinks to the 14–15- and 16–19-year-old age groups. Importantly, previous research has shown that junior athletes often start physical activity in a dehydrated state and do indeed fail to consume adequate fluid during activity [15,16]. This further highlights the need for education for parents and teens, which could address both appropriate types of fluids and fluid requirements before, during and after a sporting event or activity.

A ‘water-only’ policy for triathlon events was generally well-accepted by parents and perceived positively. Parents with children in older age groups were the least supportive of a water-only policy, and as discussed, also provided the greatest amounts of sports drinks. It is important to note that of those who were not directly supportive, a large portion were ‘unsure’, suggesting that further education and messaging would be appropriate, particularly as junior athletes grow and progress into teenage age group categories. A ‘water-only’ policy for all sporting events (adults and children) was not well-supported. This is not surprising, and there are other, existing strategies to promote water as the beverage of choice in adult sporting events and environments [17] that are likely to be more acceptable and feasible than water-only for events that include adults. Depending on the type of adult events, sports drinks may be required for reasons other than hydration (e.g., provision of carbohydrates for endurance events). Therefore, some of the resistance to support a water-only adult event policy may be due to the awareness of additional carbohydrate requirements for adult endurance sporting events.

The limitations of this study are that a cross-sectional convenience sample was obtained. Additionally, there was no demographic information obtained about the parents completing the questionnaire, such as age, education level, sex, ethnicity, sociodemographic information and experience in sport. Future work needs to consider this demographic information as parents play a vital role in supporting children’s involvement in sporting activities. Importantly, for this study the questionnaire was kept as brief as possible to ensure maximum engagement and reach at the one junior event and to gain broad attitudes and opinions from parents, as it was completed during the event when children were present/competing. However, it must also be noted that there is a risk of bias in the responses as parents may have recorded what they felt were correct, desirable or socially acceptable responses. To manage some of this bias, ‘not sure’ was added as an option. Additionally, the questionnaire was administered at a single junior event within one state of Australia. Despite this, it still provides valuable pilot data to warrant further investigation of parental attitudes towards a water-only policy and shows overall positive attitudes towards a potential water-only policy at junior sporting events. However, at this stage, there are not enough data to support creating such a policy. The current study considered opinions and attitudes of parents only. Conducting more widespread research at junior events across Victoria (and more widely, Australia) would be required to further understand parental and athlete views regarding a water-only policy for junior events. This future research should ideally extend beyond attitudes and opinions of parents and objectively assess dietary patterns and behaviours of junior athletes at these events.

## 5. Conclusions

A ‘water-only’ policy for junior sporting events could be a potential method to encourage water as the beverage of choice for children and was viewed positively by the majority of parents surveyed at the junior triathlon event. This study provides initial findings for the potential development of a water-only policy at junior triathlon events to assist in creating behaviour change and encouraging water as the beverage of choice. Whether the same positive opinion towards a water-only policy is true of parents from other sporting codes remains to be known and requires further research.

## Figures and Tables

**Table 1:** Event and age group categories at the junior triathlon competition held in Melbourne, Victoria, Australia.

Event and Distances for Each Leg of Triathlon	Age Groups
Kids Triathlon	
75 m swim, 3 km bike, 500 m run	7 to 9 year olds
200 m swim, 5 km bike, 1 km run	10 to 14 year olds
Mini Triathlon	
250 m swim, 8 km bike, 2 km run	12 to 17 year olds
Sprint Triathlon	
500 m swim, 16 km bike, 5 km run	14 to 15 year olds
500 m swim, 16 km bike, 5 km run	16 to 19 year olds

**Table 2:** Questionnaire administered to parents at the Triathlon Victoria event held in Melbourne.

Category	Questions
Current practices	For the following questions, respondents were able to select from the following options: Sports drink (e.g., Gatorade, Powerade or self-mixed)/Low or no sugar sports drink/Soft drink (e.g., Coke, Fanta, Sprite, Pepsi)/Low or no sugar soft drink (e.g., Coke Zero, Diet Coke, Pepsi Max)/Milk/Fruit Juice/Water/Other (please specify) What drink do you usually provide for your child PRIOR to them competing at a triathlon event (e.g., the morning of)? What drink do you usually provide for your child DURING a triathlon event? What drink do you usually provide for your child AFTER a triathlon event?
Current knowledge and previous education	Active children need to drink enough fluids Juice is the best option (Agree/Disagree/Not Sure) Sports drink is the best option (Agree/Disagree/Not Sure) Water is the best option (Agree/Disagree/Not Sure) During short events, you don’t really need to worry about children drinking fluid (Agree/Disagree/Not Sure) After an event, children should be encouraged to have a sports drink (Agree/Disagree/Not Sure) After an event, children should be encouraged to have cordial (Agree/Disagree/Not Sure) After an event, children should be encouraged to have a drink of water (Agree/Disagree/Not Sure) After an event, children should be encouraged to have whatever they want as they have burnt a lot of energy and they need to refuel (Agree/Disagree/Not Sure) Have you seen/received any information regarding the hydration requirements of children at sport events? (Yes/No) Did this information lead to you making any changes to the fluid you provided to your child during/after triathlon events? (No, no changes/No changes but confirmed what I was doing was right/Changes from sports drink to water/Thinking about changing in the future)
Importance of hydration	For the following questions, respondents were asked to indicate how important they think each statement is (0–10 Likert scale, where 0 = not at all important to 10 = extremely important). Please indicate how important you think it is for: Young athletes to hydrate properly PRIOR to sport? Young athletes to hydrate properly DURING sport? Young athletes to hydrate properly AFTER sport?
Opinions about a ‘water-only’ policy	For the following questions, respondents were asked to indicate how they would feel about the following (0–10 Likert scale, where 0 = strongly against, 5 = neither against nor support, 10 = strongly support): A “water-only” policy at all junior triathlon events A “water-only” policy at all junior sporting events A “water-only” policy at all triathlon events (adult and junior)

## Data Availability

The data presented in this study are available upon request from the corresponding author. The data are not publicly available due to ethical approval.
